# Incomplete Recovery from the Radiocontrast-Induced Dysregulated Cell Cycle, Adhesion, and Fibrogenesis in Renal Tubular Cells after Radiocontrast (Iohexol) Removal

**DOI:** 10.3390/ijms241310945

**Published:** 2023-06-30

**Authors:** Hsing-Yu Chen, Yi-Hong Wu, Cheng-Yu Wei, Zhi-Yao Liao, Hsiao-Ting Wu, Yung-Chang Chen, Jong-Hwei S. Pang

**Affiliations:** 1 Graduate Institute of Clinical Medical Sciences, College of Medicine, Chang Gung University, Taoyuan 33302, Taiwan; 8705016@cgmh.org.tw (H.-Y.C.); changeyu519@gmail.com (C.-Y.W.); yeats1122@hotmail.com (Z.-Y.L.); sirus1201@gmail.com (H.-T.W.); 2 Division of Chinese Internal Medicine, Center for Traditional Chinese Medicine, Chang Gung Memorial Hospital, Taoyuan 33378, Taiwan; mzpjih@gmail.com; 3 School of Traditional Chinese Medicine, College of Medicine, Chang Gung University, Taoyuan 33302, Taiwan; 4 School of Medicine, College of Medicine, Chang Gung University, Taoyuan 33302, Taiwan; cyc2356@gmail.com; 5 Division of Nephrology, Department of Internal Medicine, Chang Gung Memorial Hospital, Taoyuan 33342, Taiwan; 6 Department of Physical Medicine and Rehabilitation, Chang Gung Memorial Hospital, Taoyuan 33342, Taiwan

**Keywords:** acute kidney injury, contrast-induced nephropathy, cell cycle arrest, iohexol, polyploid cells, radiocontrast removal

## Abstract

Contrast-induced nephropathy (CIN) is one of the most common causes of acute kidney injury (AKI). However, management is still limited, and the cellular response to radiocontrast removal for CIN remains unclear. This study aimed to explore the latent effects of iohexol in cultured renal tubular cells with or without the removal of iohexol by medium replacement. HK2 renal tubular cells were subcultured 24 h before use in CIN experiments. Three treatment groups were established: the control, a radiocontrast (iohexol)-only group at 75 mg I/mL (I-75), and iohexol exposure for 24 h with culture medium replacement (I-75/M). Cell cycle arrest, fibrogenic mediator assays, cell viability, cell function, and cell-cycle-related protein expression were compared between groups. Iohexol induced numerous changes in HK2 renal tubular cells, such as enlarged cell shape, cell cycle arrest, increased apoptosis, and polyploidy. Iohexol inhibited the expression of cyclins, CDKs, ZO-1, and E-cadherin but conversely enhanced the expression of p21 and fibrosis-related genes, including TGF-β1, CTGF, collagen I, collagen III, and HIF-1α within 60 hr after the exposure. Except for the recovery from cell cycle arrest and cell cycle gene expression, notably, the removal of iohexol by medium replacement could not fully recover the renal tubular cells from the formation of polyploid cells, the adhesion or spreading, or the expression of fibrosis-related genes. The present study demonstrates, for the first time, that iohexol exerts latent cytotoxic effects on cultured renal tubular cells after its removal, suggesting that these irreversible cell changes may cause the insufficiency of radiocontrast reduction in CIN, which is worth investigating further.

## 1. Introduction

Contrast-induced nephropathy (CIN) is the third leading cause of acute kidney injury (AKI) during hospitalization. It has become a severe problem for the healthcare system due to the high prevalence of procedures requiring contrast media (CM) [1,2]. Among patients with diabetes mellitus (DM), the incidence of CIN could be as high as 9%, while it could be 90% among patients with both DM and chronic kidney disease (CKD). In addition, for patients undergoing interventional and diagnostic coronary angiography, the incidence is 15% and 1.6–2.3% [3,4,5]. With the increased use of CM-dependent examinations in recent decades, CIN will become a more severe problem in the future [6]. Moreover, the severity of CIN largely depends on CM type and exposure duration [7,8]. Different CMs can vary in their chemical composition, osmolality, and viscosity, which may influence their potential to cause kidney injury. The two main categories of contrast agents are high-osmolality contrast media (HOCM) and low-osmolality contrast media (LOCM). Additionally, newer types of contrast agents, such as iso-osmolar contrast media (IOCM) and low-osmolality, iso-osmolar contrast media (LOCM-IOCM), have been developed to potentially minimize the risk of CIN [7,8]. Some studies have reported a lower incidence of CIN with LOCM, IOCM, or LOCM-IOCM than HOCM [7]. For this reason, iohexol has been commonly used in clinical practice to avoid CIN as a kind of LOCM; however, a substantial proportion of subjects still suffer from CIN after using iohexol [9,10]. The duration of CM exposure can vary depending on the specific experimental design and research objectives. In many in vitro studies, exposure durations range from 6 to 24 h to assess immediate cytotoxic effects or early cellular responses [11,12,13,14]. Nevertheless, studies about the latent cytotoxic effect of CM exposure are still lacking, even though long-term renal damage could be observed in the clinical setting [15].

Several studies have demonstrated the possible mechanisms of CIN, including poor renal perfusion, disturbed renal tubuloglomerular feedback, direct renal cell damage due to hypoxia, and reactive oxygen species (ROS) generation [2]. Although not reabsorbed by renal tubular cells, CM may cause renal tubular cell damage regardless of hemodynamic changes [16]. Cell cycle arrest, an essential response to other types of AKI, has, however, not yet been reported in response to the administration of CM. The role of cell cycle arrest in AKI has become an exciting field of study in recent years, including its role in reperfusion-ischemia injury (IRI), unilateral nephrectomy, and cisplatin-induced nephropathy [17,18,19,20]. However, cell cycle arrest in CIN has not been well demonstrated. On the other hand, in most studies exploring the management of CIN, only the acute effects of CM exposure on renal cells have been investigated without anticipating the latent impact of CM after the removal and volume expansion commonly associated with CIN. Since personalized management for CIN is requested, it is essential to explore the cellular response to CIN with consideration of clinical settings [2]. For example, for patients unsuitable for volume expansion or immediate CM removal after examination, prolonged CM exposure and following latent impact should be managed individually to avoid potential side effects of volume expansion therapy [21,22].

This study aims at assessing the immediate and latent changes in cell cycle, adhesion ability, and fibrogenesis in renal tubular cells in CIN with and without CM removal. For this purpose, we established an in vitro model that mimics clinical practices and prolongs the observation period for possible latent effects of CIN. Understanding the cell responses to CIN with possible clinical scenarios would broaden the horizon of managing CIN.

## 2. Results

### 2.1. Irreversible Iohexol-Induced Changes in Renal Tubular Cells after Its Removal

**Figure 1:**
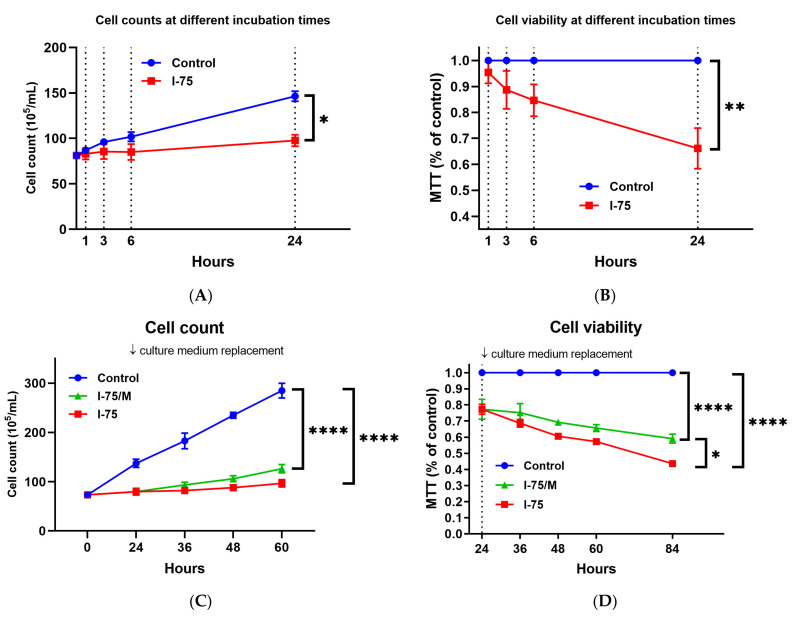
The cytotoxic effect of iohexol on HK2 renal tubular cells could not be fully reversed by culture medium replacement. Cell numbers (**A**,**C**) and viability (**B**,**D**) in different groups at the indicated time points were counted with a trypan blue exclusion assay and measured with an MTT assay, respectively. Three independent experiments were carried out to present these results. Values are mean ± standard deviation. Statistical analysis by two-way repeated ANOVA; * *p*-value < 0.05, ** *p*-value < 0.01, **** *p*-value < 0.0001.

To investigate the cytotoxicity of iohexol on renal tubular cells, 75 mg I/mL of iohexol was added to cultured cells 24 h after subculture, and cell number and cell viability were examined in three treatment groups as described. Increases in cell number and cell viability were quickly suppressed within 3 h of iohexol exposure (Figure 1A,B, respectively). Removing iohexol by replacing culture media 24 h after iohexol exposure did not eliminate the suppressive effects of iohexol (Figure 1C, *p* < 0.001). Similar results were obtained by measuring cell viability using an MTT assay, as shown in Figure 1D (*p* < 0.001). Only slight differences in cell number and viability were observed between cells in the I-75 and I-75/M groups (Figure 1C,D; *p* = 0.13 for cell number and *p* = 0.039 for cell viability).

**Figure 2:**
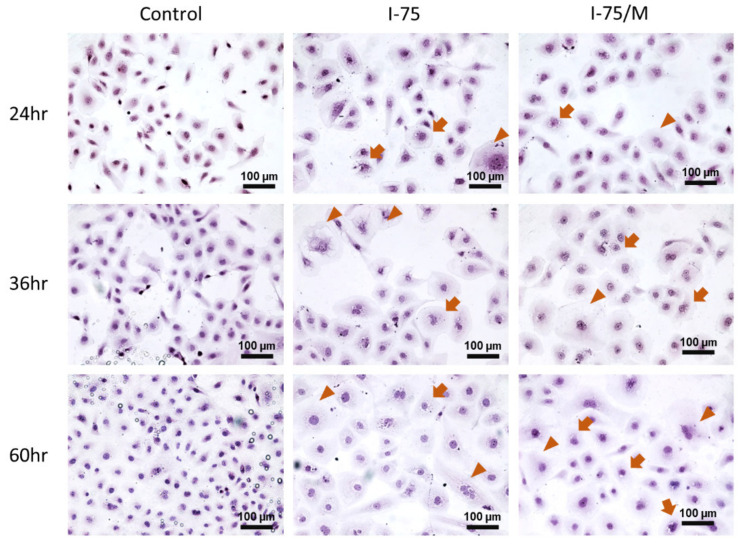
The changes in cell morphology revealed the remaining cytotoxic effect of iohexol on HK2 renal tubular cells after removing iohexol. The morphology and nuclei of HK2 cells were revealed by hematoxylin and eosin staining and imaged with a light microscope. Arrows indicate cells with vacuolization, and arrowheads indicate large polyploid cells.

HK2 cells became flattened and enlarged after adding iohexol, and then vacuoles formed in the cytosols. The number of polyploid cells increased with time and slightly even after removing iohexol (Figure 2, arrows indicating cells with vacuolization and arrowheads showing large polyploid cells). Similar morphological changes were found in NRK52E cells, another commonly used renal tubular cell line derived from rats (Supplementary Figure S1). To investigate whether HK cells could recover after an extended period, cells were observed for up to 120 h after medium replacement. Notably, the large polyploid cells remained in culture even for an extended period after the iohexol removal (Supplementary Figure S2).

### 2.2. Removal of Iohexol Reduced Apoptosis but Slightly Increased G2/M Arrest and Polyploidy

**Figure 3:**
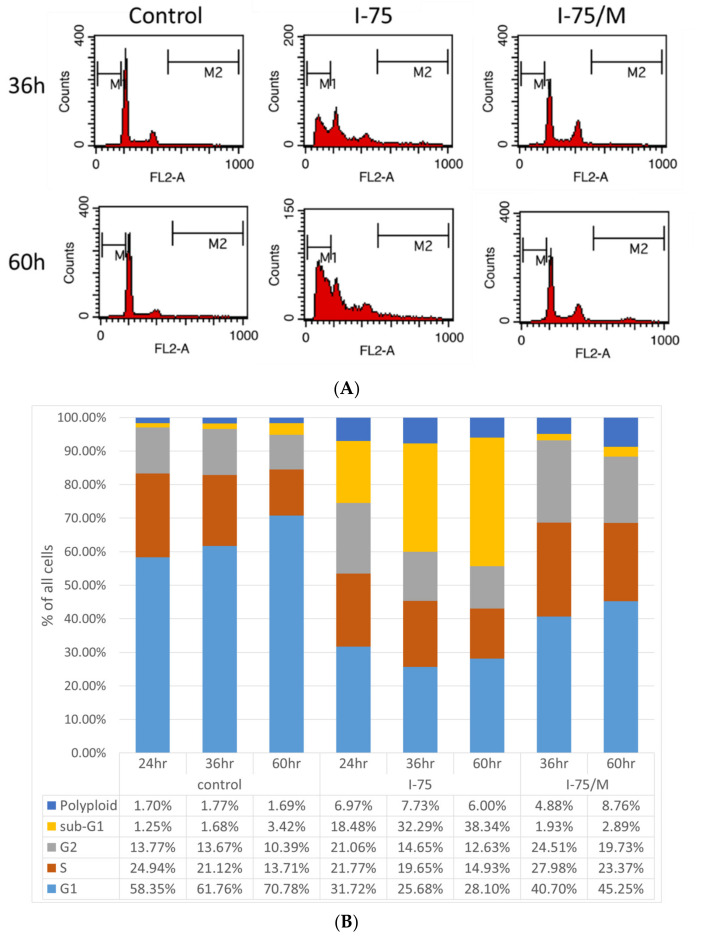
Removal of iohexol did not fully reverse the changes in the cell cycle state in renal tubular cells. (**A**) Changes in cell cycle state in different groups at the indicated time points were analyzed by flow cytometry. (**B**) The average fraction of each cell cycle in different groups at the indicated time points was calculated from three independent experiments. The measurement of cell proportion on 24 h I-75/M was as exact as 24 h I-75 cells and therefore was not shown in this figure.

Our flow cytometric analysis of the cell cycle state demonstrated that iohexol induced a significant increase in subG1 apoptosis in a time-dependent manner, which then returned to a similar level to the control after removing iohexol. This could be due to removing most apoptotic cells after replacing the culture medium (Figure 3A,B). Iohexol treatment significantly decreased cells in the G1 phase, which was partially reversed after removing iohexol. The subtle changes caused by iohexol, including a reduction in the S phase and an increase in G2/M phase cells, suggest that the loss of cells through apoptosis might occur mainly in G1 cells (Figure 3A,B). Unexpectedly, iohexol treatment markedly increased the number of polyploid cells, which remained high compared to control cells and increased further even after removing iohexol (Figure 3A,B).

**Figure 4:**
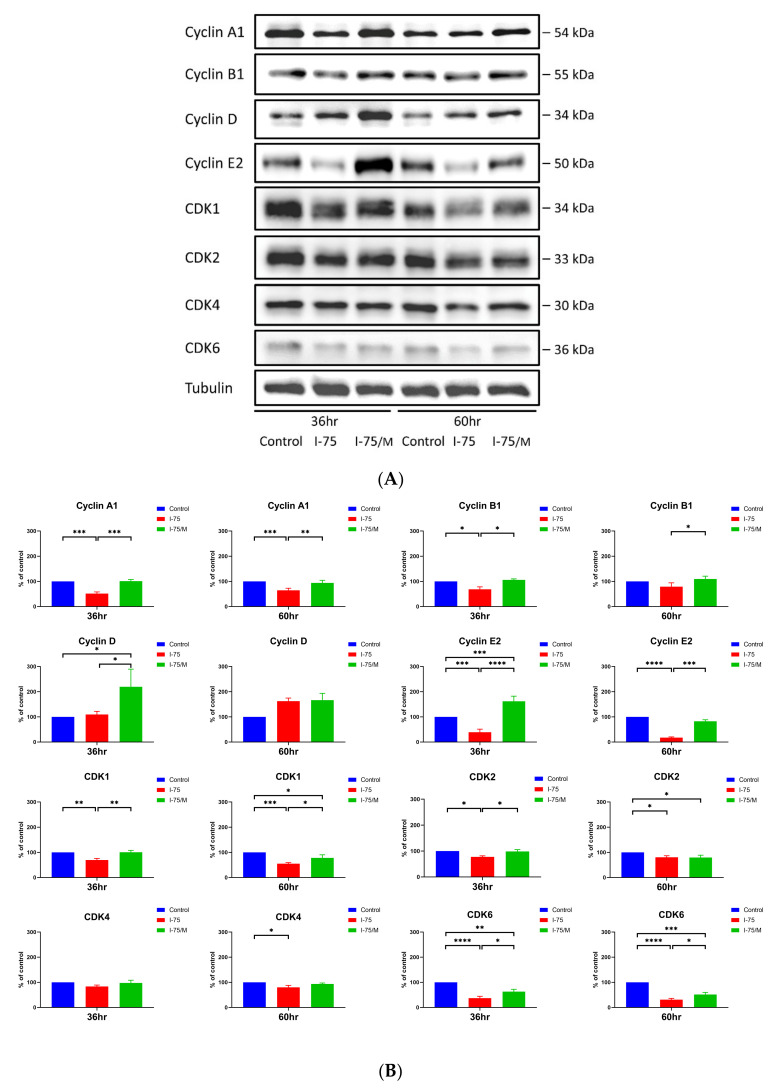
Replacement of culture medium reversed the effect of iohexol on the expression of most cell-cycle-dependent proteins except cyclin D, CDK2, and CDK6. (**A**) Protein extracts were prepared from renal tubular cells in different groups at the indicated time points and processed for Western blotting analysis. (**B**) Quantitative analysis of the expression levels of cell-cycle-dependent proteins at the indicated time points was calculated from three independent experiments. Values are mean ± standard error of the mean. Statistical analysis by two-way ANOVA with Sidak’s post hoc test; * *p*-value < 0.05, ** *p*-value < 0.01, *** *p*-value < 0.001, **** *p*-value < 0.0001.

Moreover, the increase in polyploid cells remained elevated at 60 h after the removal of iohexol, suggesting a sustained effect that could not be reversed. Most cell-cycle-related cyclins and CDKs were suppressed when HK2 cells were exposed to iohexol. However, iohexol did not significantly change the expression of cyclin D and CDK4 after 36 h of treatment (Figure 4A,B) but did increase cyclin D expression after 60 h of treatment. Notably, the expression of cyclin D was further enhanced even after removing iohexol, corresponding to the increase in polyploidy in I-75/M cells. Among the iohexol-induced decreases in cyclin A, E, B, CDK1, 2, and 6, only CDK2 and 6 could not be fully reversed after its removal (Figure 4A,B).

### 2.3. Effect of Medium Replacement on the Expression of p21, p27, and p53 after Iohexol Treatment

**Figure 5:**
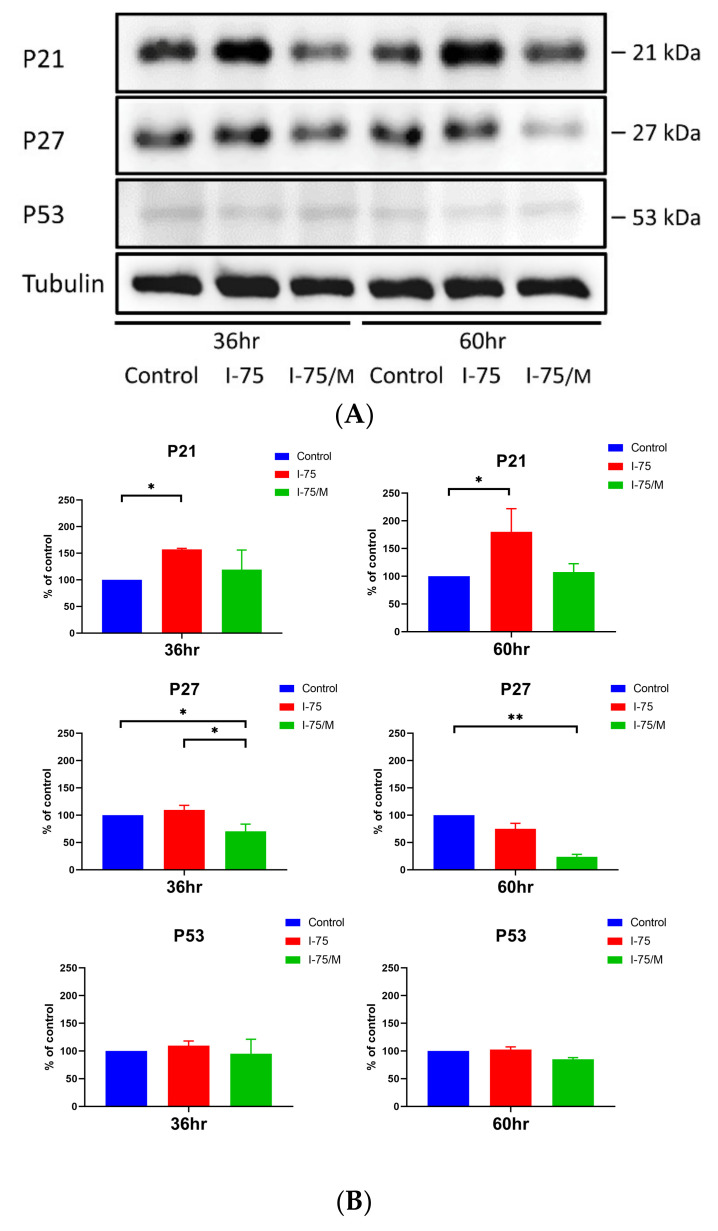
Effect of iohexol on the expression of cell cycle inhibitor proteins in renal tubular cells. (**A**) Protein extracts were prepared from renal tubular cells in different groups at indicated time points and processed for Western blotting analysis. (**B**) Quantitative analysis of p53, p21, and p27 expression levels at indicated time points was calculated from three independent experiments. Values are mean ± standard error of the mean. Statistical analysis by two-way ANOVA with Sidak’s post hoc test; * *p*-value < 0.05, ** *p*-value < 0.01.

Iohexol upregulated the protein expression of p21, but not p27 or p53, in renal tubular cells. The removal of iohexol from the culture medium reversed the increase in P21 protein back to the level of the control group. Interestingly, removing iohexol significantly reduced the protein expression of p27, which might contribute partially to the sharp decrease in apoptosis observed after replacing culture media (Figure 5A,B). On the other hand, p53 remained unchanged under conditions of iohexol exposure and removal.

### 2.4. Iohexol Impaired the Adhesion, Spreading, and Expression of ZO-1 and E-cadherin in Renal Tubular Cells

**Figure 6:**
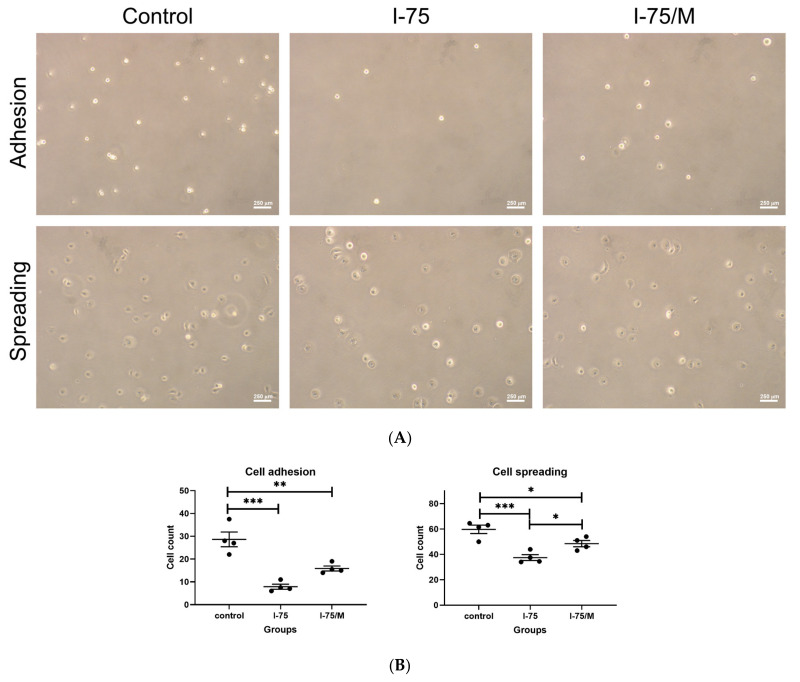

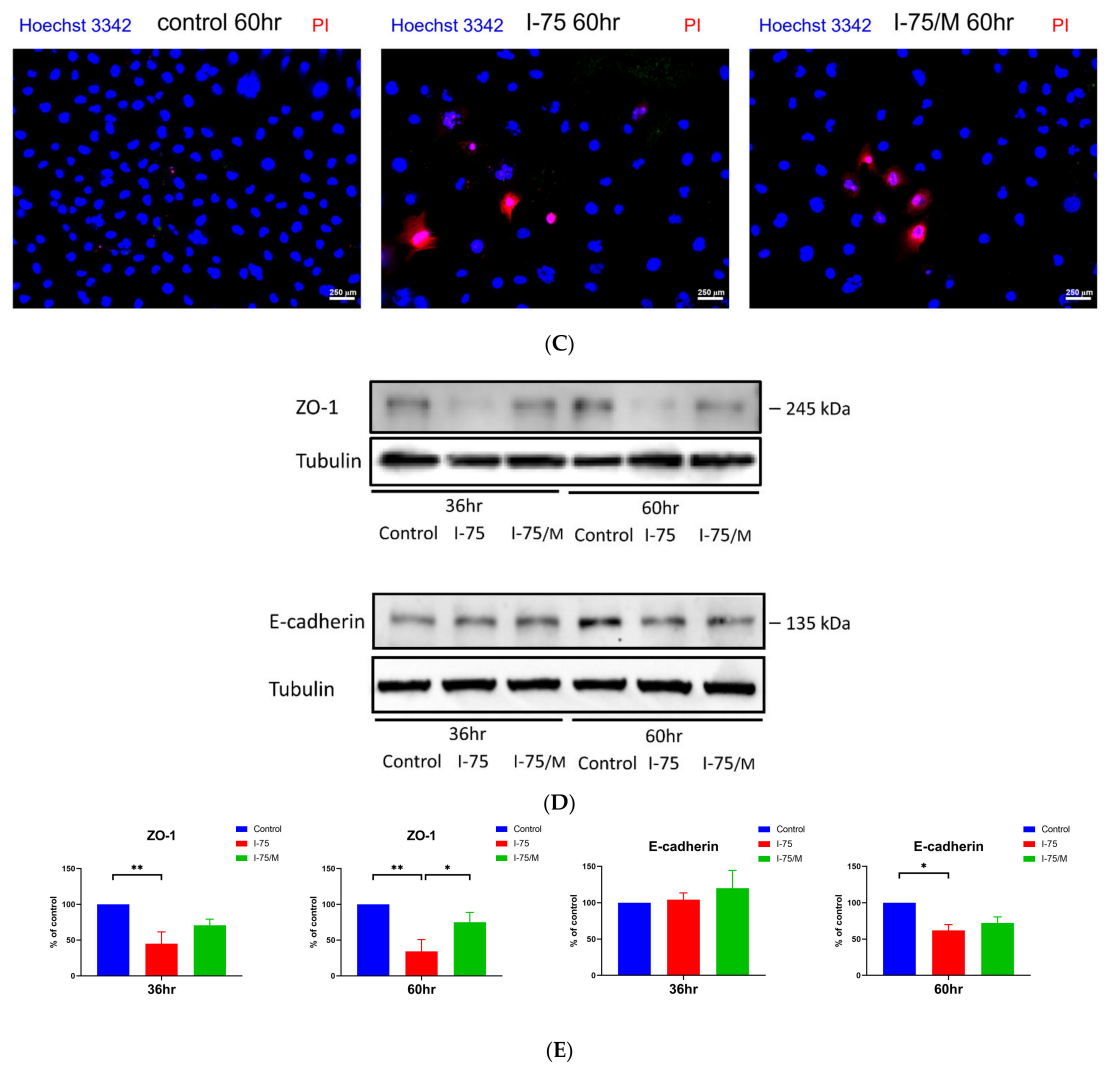
The iohexol-induced suppression of cell adhesion and spreading and expression of ZO-1 and E-cadherin in renal tubular cells could not be recovered fully after medium replacement. (**A**) Renal tubular cells prepared from three different groups at indicated time points as described were processed for the analysis of adhesion and spreading by observing cell behavior immediately after subculture. (**B**) Data were quantified from four independent experiments. (**C**) Cells from three treatment groups at 60 h after medium replacement were stained by PI without prior fixation and observed using a fluorescent microscope. Cytosolic red fluorescence confirmed the damage to membrane integrity. (**D**) Protein extracts were prepared from renal tubular cells in different groups at indicated time points and processed for Western blotting analysis to examine the expression of ZO-1 and E-cadherin. (**E**) Quantification of ZO-1 and E-cadherin expression levels was calculated from three repeated experiments. Values are mean ± standard error of the mean. Statistical analysis by two-way ANOVA with Sidak’s post hoc test; * *p*-value < 0.05, ** *p*-value < 0.01, *** *p*-value < 0.001.

Our results demonstrated that the adhesion and spreading of renal tubular cells were markedly impaired after iohexol exposure, and the recovery after its removal, especially for cellular adhesion, was limited in the I-75/M group (Figure 6A,B). PI staining was performed on renal tubular cells in the three different groups at the indicated time to reveal the effect of iohexol on membrane integrity. PI is a red membrane-impermeable fluorescent dye that only enters cytosol when cell membrane integrity is damaged. The presence of red fluorescence in cytosol indicated that iohexol resulted in cell membrane damage, which could not be reversed by its removal (Figure 6C). We also found that the PI stain tended to co-exist with large polyploid cells in I-75 and I-75/M cells. The expression of ZO-1 and E-cadherin was investigated at the indicated time points since they are tight junction proteins involved in cell membrane integrity and permeability. Iohexol suppressed the expression of ZO-1 earlier than that of E-cadherin, which was not affected until 60 h post-treatment. However, the removal of iohexol did not eliminate its inhibitory effect on the reduced expression of either ZO-1 or E-cadherin (Figure 6D,E).

### 2.5. Iohexol Removal Did Not Fully Reverse the Increase in Fibrosis-Related Protein Expression

**Figure 7:**
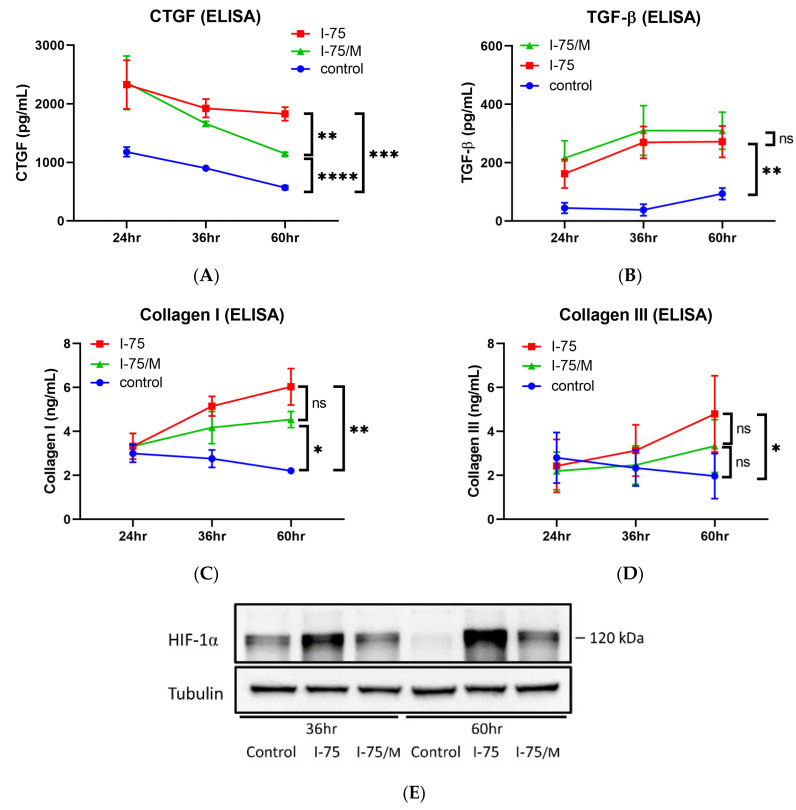

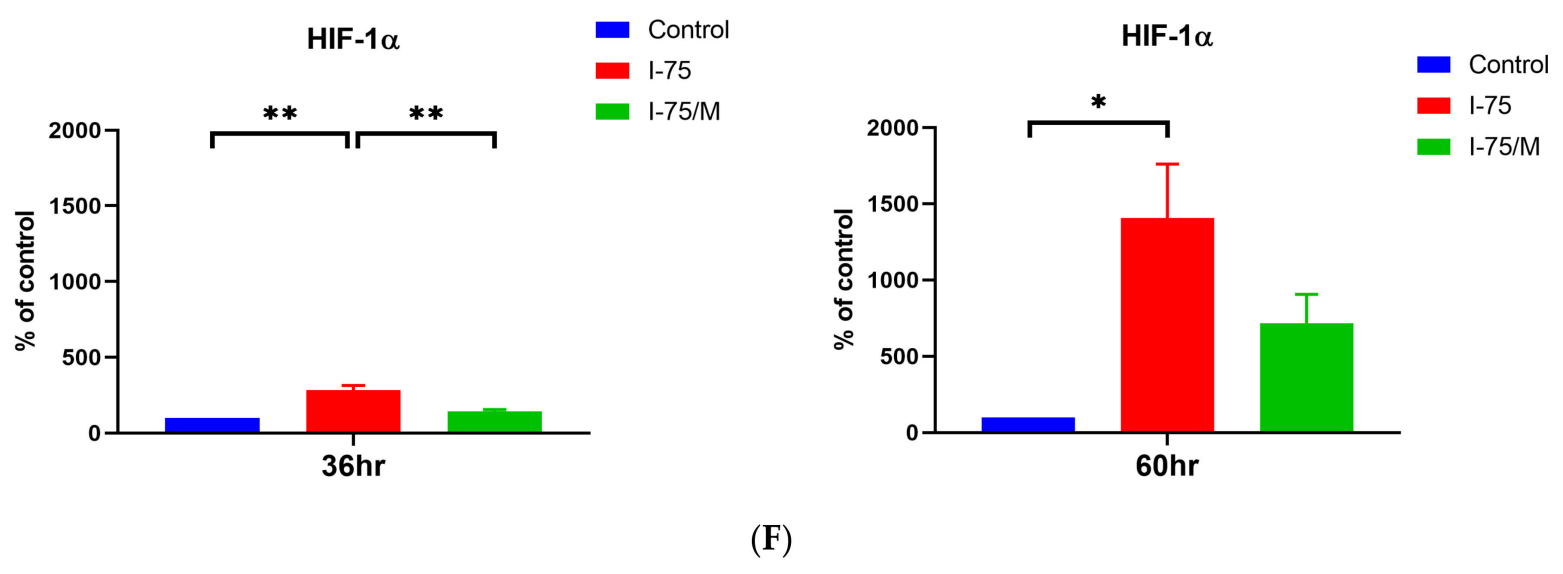
The iohexol-induced increase in fibrosis-related proteins could not be fully reversed after medium replacement. Conditioned medium collected from three treatment groups at the indicated time points was used to determine the levels of (**A**) CTGF, (**B**) TGF-β, (**C**) collagen I, (**D**) and collagen III by ELISA; (**E**,**F**) HIF-1α expression was confirmed by Western blot analysis. Three independent experiments were carried out to quantify these results statistically. Values are mean ± standard error of the mean. Statistical analysis by two-way ANOVA with Sidak’s post hoc test; ns *p*-value > 0.05, * *p*-value < 0.05, ** *p*-value < 0.01, *** *p*-value < 0.001, **** *p*-value < 0.0001.

To examine the effect of iohexol on the expression of fibrosis-related proteins in renal tubular cells, the levels of CTGF, TGF-β, collagen I, and collagen III in a conditioned medium were measured by ELISA (Figure 7A–D). Iohexol significantly enhanced the expression of CTGF, which reached a plateau at 24 h and declined after removing iohexol. The protein levels of TGF-β, collagen I, and collagen III increased in a time-dependent manner up to 60 h. The amount of TGF-β remained unchanged after removing iohexol. The amount of CTGF, collagen I, and collagen III was reduced in the I-75/M group compared to the I-75 group. However, CTGF, TGF-β, and collagen I levels, but not collagen III, were still much higher than in the control group (Figure 7A–D). The expression of HIF-1α, which is involved in the early signaling process of fibrogenesis, was significantly induced in renal tubular cells by iohexol. HIF-1α expression was highest at 60 h after iohexol exposure (Figure 7E,F). Moreover, although the level of HIF-1α decreased after culture medium replacement, the level of HIF-1α remained much higher at 60 h in the I-75/M group compared to that in the control group.

## 3. Discussion

In this study, we established an in vitro model of CIN to examine the cellular changes in CIN with and without CM removal. This is the first study addressing CIN’s advantages and potential adverse influences with CM removal. Most studies about the reno-protective mechanisms of hydration focused on removing CM, increasing perfusion over the renal medulla, and diluting CM in renal tubules, which was supposed to benefit renal tubule cells from CIN by shortening exposure time to CM [23,24]. The previous literature has extensively discussed the effects and pathogenesis of multiple types of CM; however, the effects of hydration, the most commonly used management tool in a clinical setting, have seldom been examined at the cellular level. Consequently, the role of hydration in managing CIN has been questionable for years [2,21,22,25,26]. In this study, we demonstrate the advantages of CM removal: cell recovery did occur after CM removal, a substantial proportion of HK2 cells reverted to control-like cells, and arrested cell cycles seemed to restore progress. These in vitro findings provide essential evidence for the beneficial effect of hydration commonly used to remove CM clinically.

This in vitro cell model with CM being removed by medium replacement may also be useful for exploring potential treatments for CIN since previous studies only focused on the effect of medication compared to no treatment [27,28,29,30,31,32]. However, as the standard management in clinical settings, hydration has seldom been used as a control group in laboratory studies [11,33,34,35,36]. CIN was initially regarded as a spontaneous recovery process among healthy people. This recovery capability may, however, also be necessary when repeated angiography or other CM-based examinations are needed, as well as in patients who cannot have adequate hydration or experience acute illnesses [37,38]. The recovery rate may be crucial for arranging further CM-dependent examinations or even preventing latent kidney complications, given that the total recovery rate may be small among acutely ill patients [38]. Therefore, using this CM-removal model with observation of the latent effect of CIN in screening medications for CIN may be more practical and closer to the clinical setting.

Our study extended the observation period to 60 h after iohexol exposure and found that the effects of iohexol removal may be limited in several aspects that were seldom observed in previous studies, which focused only on 6–24 h after exposure [11,12,13,14]. The extended observation period for the in vitro CIN model may be necessary due to the potential delayed effects of CIN found in clinical practice and the possible opposing effects of short- and long-term cell cycle arrest [15,39,40,41]. The typical presentation of CIN, including cytosolic vacuolation and apoptosis, has been demonstrated in renal tubular cells persistently exposed to iohexol [42,43]. At the cellular level, we provide evidence that the cell cycle was arrested at the G2/M phase, which has not been reported in CIN as opposed to other types of renal injury [18,19,44]. As the exposure time of iohexol became longer, the cell cycle arrest may have been accompanied by increasing numbers of apoptotic cells, polyploid cells, poor residual cell function, and worsening fibrosis. The fate of arrested cells in the iohexol-only group (I-75) is likely cell death since the proportion of apoptotic cells increased dramatically as the number of polyploid and arrested cells decreased. Indeed, the decrease in markers of cell function and increase in fibrogenic markers support this hypothesis. Cell cycle arrest in G2/M may be associated with cells with large nuclei, contributing to a shorter life span for these cells [41,44,45]. Cell cycle arrest was reported to be a response to CKD and AKI; however, the subsequent fate of arrested cells may differ depending on the type and duration of the injury. Yang et al. reported that only severe renal injuries, such as severe IRI, acute aristolochic acid toxic nephropathy, and ureteral obstruction, can lead to overt renal injury with G2/M arrest and subsequent renal fibrosis [44]. However, Wen et al. reported that short-term cell cycle arrest might improve acute renal injury and that only sustained maladaptive proximal renal tubular cells may result in renal fibrosis [40,46]. Our model of CIN mimicked severe renal injury, in which persistent G2/M arrest with a low percentage of S phase cells and progressively poor cell outcomes were found. This finding contributes to our knowledge about the relationship between cell cycle arrest and the outcomes of CIN.

Despite the potential benefit of culture medium replacement, our data strongly suggest that interventions for residual malfunctioning cells may still be needed after hydration. This may also partially explain the controversy regarding the hydration-only management of CIN [21,22,25,26]. The arrested cell cycle seemed to recover in a large proportion of cells, reflected by the increasing proportion of S phase cells in the I-75/M group and the changes in the expression of cyclins and CDKs. Moreover, the increased cyclin D level persisted after culture medium replacement, corresponding with the increased proportion of the G1/S phase of cells. This may lead to the elevated formation of polyploid cells in the I-75/M group [47]. Overall cell adhesion, spreading, and the increasing trend of fibrogenic factors appeared to be partially improved after CM removal. Nonetheless, based on our continuous observation of cell viability and morphology, we found the fate of residual cells after iohexol exposure was heterogeneous. With the release of arrested cell cycles, some cells became apoptotic or polyploid. Since dead and unhealthy cells were removed along with iohexol when replacing the culture medium, the dead cells found at 36 and 60 h in the I-75/M group were presumably generated de novo. The delayed effects of CIN may correspond to a recent interpretation of AKD, which calls attention to potentially unrecovered renal cells after AKI that may become potential management targets [48].

The increasing proportion of polyploid cells in both I-75 and I-75M groups may be another treatment target, especially because high cyclin D levels are commonly correlated with carcinogenesis based on higher polyploid formation and resistance to apoptosis [49]. However, the function of polyploid cells is still largely undetermined. For example, the appearance of polyploid cells indicated cell regeneration, de-differentiation, and subsequent proliferation in a hepatocyte model [50]. Nevertheless, the induction of polyploid cells in another study was also related to cell cycle arrest and further DNA instability in carcinogenesis [51]. Since the role of polyploid cells in CIN is poorly defined, our data provide evidence that iohexol causes the substantial formation of polyploid cells, which are commonly large cells. More interestingly, these cells seemed quite sensitive to signals from surrounding cells that triggered the process of apoptosis (presented in supplementary Video S3A,B). This finding may also further reveal the role of polyploid cells with large nuclei in CIN during hydration therapy. For these reasons, the cell cycle and polyploid cells may serve as novel targets for managing CIN even when hydration is provided [52,53].

HIF-1α and p21-related pathways may be the critical mediators of cell cycle arrest and polyploid cells in CIN, but the roles of HIF-1α and p21 have been scarcely studied. The management of the pathogenesis of CIN at the cellular level is mainly based on the hypothesis that hydration for CIN could flush out damaged tubular cells and restore homeostatic pH levels, reduce reactive oxygen species (ROS) generation, and revert osmotic changes during CIN [23,54]. Cellular cytoskeletons may have changed following these events, and cell morphology and function, such as migration and spreading, may have become subsequently altered [55]. Furthermore, HIF-1α, an external-stress-related protein, is related to downstream ROS production in other kinds of renal injuries. HIF-1α overexpression may also lead to increased TGF-β levels and subsequent pro-fibrotic changes, like the ones we found in our CIN model [56,57]. P21 is thought to be the critical mediator of cell cycle arrest in renal fibrosis, and its activation was found to be associated with the induction of HIF-1α. However, the role of HIF-1α may vary in different renal injuries [58,59]. In our CIN model, we found that the changes in p21 and HIF-1α expression were different between I-75 and I-75/M groups. The expression of HIF-1α decreased rapidly after culture medium replacement while remaining higher than the control group. On the other hand, p21 levels recovered at only 60 h post-treatment in the I-75/M group and were expressed in cells with relatively large nuclei in both I-75 and I-75/M groups. This implies that another mediator may retain p21 in these cells and cause cell cycle arrest or polyploidy in a substantial proportion of residual cells after exposure to iohexol. This may also be the reason why fibrogenic proteins were still high in the I-75/M group, which is led by p21-dependent G2/M cell cycle arrest in the early-stage renal fibrosis in the ureteral obstruction model [60].

## 4. Materials and Methods

### 4.1. Cell Culture and Experiment Model for CIN

**Figure 8:**
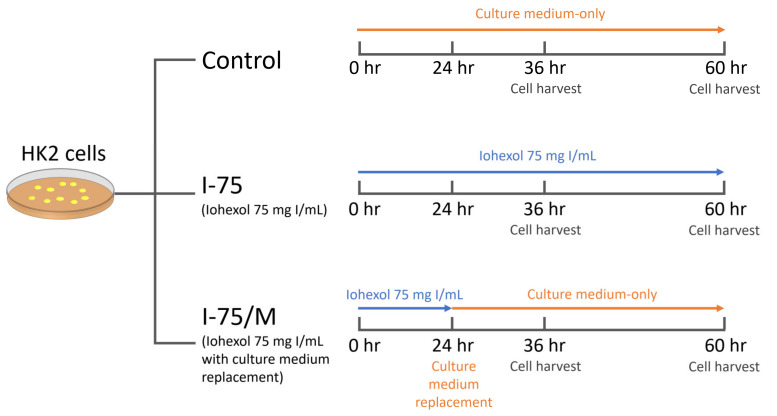
The diagram shows the experimental design of the in vitro CIN model.

Renal proximal tubular cells, including HK2 (human) and NRK52E (rat), commonly used for in vitro experiments of kidney study, were obtained from ATCC [44,61,62,63]. Cells were cultured in RPMI 1640 medium containing 10% heat-inactivated FBS and 100 μg/mL streptomycin/penicillin at 37 °C in a humidified chamber with 5% CO_2_. Figure 8 shows the experimental design of the in vitro CIN model with the culture medium replacement to remove iohexol. Cells were subcultured 24 h before the addition of iohexol. Then, iohexol (Omnipaque, GE Healthcare, Chicago, IL, USA; 350 mg I/mL) at a dose of 75 mg I/mL was added to cultured cells indicated as 0 h in Figure 8. The concentration of iohexol used in this study was based on the clinical dose of 1–1.5 mL/kg body weight, leading to a plasma concentration of 15–20 mg I/mL. Assuming that about 70–80% of glomerular filtrate would be reabsorbed in the proximal convoluted tubules, the iohexol concentration would be 75–100 mg I/mL [64]. Three groups of HK2 cells were examined: the control group (culture medium only), the I-75 group (culture medium with 75 mg I/mL iohexol), and then the I-75/M group (iohexol-free culture medium replacement at 24 h after iohexol exposure). In clinical practice, the kidney excretes the CM rapidly when volume expansion therapy is feasible. At the same time, the exposure period may be extended among patients unsuitable for volume expansion therapy [64]. Therefore, the culture medium replacement was set at 24 h after the iohexol exposure period in the CM-removal group (I-75/M). Cells were harvested at 36 and 60 h to evaluate possible latent effects, as indicated in Figure 8, for the following analysis.

### 4.2. MTT Assay

HK2 cells were seeded in a 96-well plate and treated as described above. At the indicated time, cells were washed once with 1 × PBS, and then 1 mL of DMEM containing 0.05 mg/mL 3-[4,5-2-yl]-2,5-diphenyltetrazolium bromide (MTT)/well was added to the plate. The culture medium was removed after incubation at 37 °C for one hour. The formazan crystals in the cells were dissolved with 1 mL of DMSO, and OD values were determined at 570 nm using a spectrophotometer.

### 4.3. Flow Cytometric Analysis of Cell Cycle State, Apoptosis, and Polyploidy

The effect of iohexol exposure and culture medium replacement on the cell cycle state was analyzed by flow cytometry [65]. First, cells were trypsinized, fixed with 1 mL of ice-cold 70% ethanol, and incubated at −20 °C for 2 h. Next, cells were collected, washed, and incubated in 0.5% Triton X-100 containing 0.05% RNase at 37 °C for 1 h. Finally, cell nuclei were stained with 50 mg/mL propidium iodide, diluted in 1 × PBS, and incubated at 4 °C for 20 min. Cell cycle states, including sub-G1 (apoptosis), G1, S, G2/M, and polyploidy, were analyzed using a FACS Calibur system (Becton-Dickinson, Franklin Lakes, NJ, USA) and Cell Quest Pro software version 5.1 (Becton Dickinson).

### 4.4. Protein Extraction and Western Blot Analysis

Cellular protein lysates were extracted and prepared in lysis buffer (150 mM NaCl, 1 mM Na_2_EDTA, 20 mM Tris-Cl pH 7.5, 1 mM β-glycerophosphate, 1% Triton, 2.5 mM sodium pyrophosphate, 1 mM ethylene glycol-bis (β-aminoethylether)-N,N,N′,N′-tetra acetic acid (EGTA), 1 mM phenylmethylsulfonyl fluoride, 1 μg/mL leupeptin, 1 μg/mL aprotinin, and 1 mM Na_3_VO_4_), followed by centrifugation at 12,000× *g* for 20 min at 4 °C. The protein concentration in the supernatant was analyzed using the Bradford protein assay kit (Bio-Rad, Hercules, CA, USA). First, equal amounts of protein were separated by a 10% SDS-PAGE with subsequent transfer onto a PVDF membrane. Next, non-specific binding sites were blocked by incubating the membrane for 1 h with 5% non-fat dried milk in 1 × TBS at room temperature. Then, the membrane was incubated in a blocking solution containing diluted primary antibodies against ZO-1 (ARG55738, Arigo, Hsinchu City, Taiwan), E-cadherin (#3195, Cell Signaling Technology, Danvers, MA, USA), cyclin A1 (sc-271645, Santa Cruz, Dallas, TX, USA), cyclin B1 (55004-1-AP, Proteintech, Rosemont, IL, USA), cyclin D (#ABE52, Millipore, Burlington, MA, USA), cyclin E2 (ab40890, Abcam), CDK1 (ARG66281, Arigo), CDK2 (10122-1-AP, Proteintech), CDK4 (#12790, Cell Signaling Technology), CDK6 (#3136, Cell Signaling Technology), p21 (#2947, Cell Signaling Technology), or HIF-1α (#14179, Cell Signaling Technology) at room temperature for 1 h. The membrane was then washed twice with buffer solution (1 × TBS with 0.05% Tween 20) for 5 min. After washing, the membrane was incubated for 1 h in 1 × PBS containing goat anti-mouse or anti-rabbit IgG conjugated with horseradish peroxidase (Sigma, St. Louis, MO, USA). After washing, the membranes were developed with an enhanced chemiluminescence reagent (Amersham Pharmacia Biotech, Piscataway, NJ, USA). A housekeeping protein (tubulin) was used as an internal control to normalize protein quantity.

### 4.5. Cell Adhesion and Spreading

Cell behavior after the trypsinization and replating was recorded to assess the adhesion and spreading of cells at the indicated time after treatment. For the former, floating cells were washed off 10 min after replating, and adhered cells were counted with a microscope. For the latter, floating cells were washed off 30 min after replating, and the spreading of cells presenting a flattened morphology was evaluated and counted with a microscope [66,67]. The average cell count was calculated from five visual fields (100×), and this experiment was carried out in four replicates.

### 4.6. ELISA

The levels of fibrosis-related proteins, including CTGF, TGF-β, collagen I, and collagen III, were quantified in the cell culture medium. The following ELISA kits were used: CTGF (tcee701, Taiclone, Taipei, Taiwan), TGF-β (ARG80123, Arigo), collagen I (tcee737, Taiclone), and collagen III (tcee650, Taiclone). An equal amount of conditioned medium from each treatment group was used and processed using the double-antibody sandwich method according to the method described in the manufacturer’s protocol. At least three independent experiments were carried out to confirm these results.

### 4.7. Statistical Analysis

Data obtained from measurements of cell count, cell viability, CTGF, TGF-β, collagen I, and collagen III levels were examined with respect to time by two-way repeated ANOVA. In addition, a comparison of cell adhesion and spreading, as well as quantification of proteins between groups, was carried out using one-way ANOVA and post hoc pairwise two-tailed Student’s *t*-test with Welch’s correction. All statistics with a *p*-value < 0.05 were considered statistically significant.

## 5. Conclusions

In this study, we present an in vitro model of CIN to observe changes with and without iohexol removal. This may mimic the behavior of cells in CIN in the context of hydration, which has not been studied extensively. We found that not all cells reverted to control-like cells during observation, even after culture medium replacement. This finding agrees with the current ambiguous status of hydration in clinical studies. G2/M arrest and cell polyploidy remained even after hydration, and the potential negative impact of large/polyploid cells may suggest a novel treatment target. Finally, this model could be used for studying new treatment targets for CIN in the context of hydration, which may make management more practical in clinical settings. Nevertheless, the current study still lacks to explore interactions between renal tubules, endothelium, and renal interstitial tissues, although renal tubular cells are the primary injured location of CIN. We also cannot present the composite renal outcomes in the in vitro platform. For this reason, in vivo studies are still needed to prove the existence of cell cycle arrest and polyploid cells and the renal outcome of CIN with and without iohexol removal.

## Data Availability

The data presented in this study are available in the Supplementary Materials.

## Supplementary Materials

The supporting information can be downloaded at: https://www.mdpi.com/article/10.3390/ijms241310945/s1.
