# Supplementary figures and images for: Incomplete Recovery from the Radiocontrast-Induced Dysregulated Cell Cycle, Adhesion, and Fibrogenesis in Renal Tubular Cells after Radiocontrast (Iohexol) Removal
Source: Int J Mol Sci. 2023 Jun 30;24(13):10945. doi: 10.3390/ijms241310945 (PMC10341645; doi:10.3390/ijms241310945)

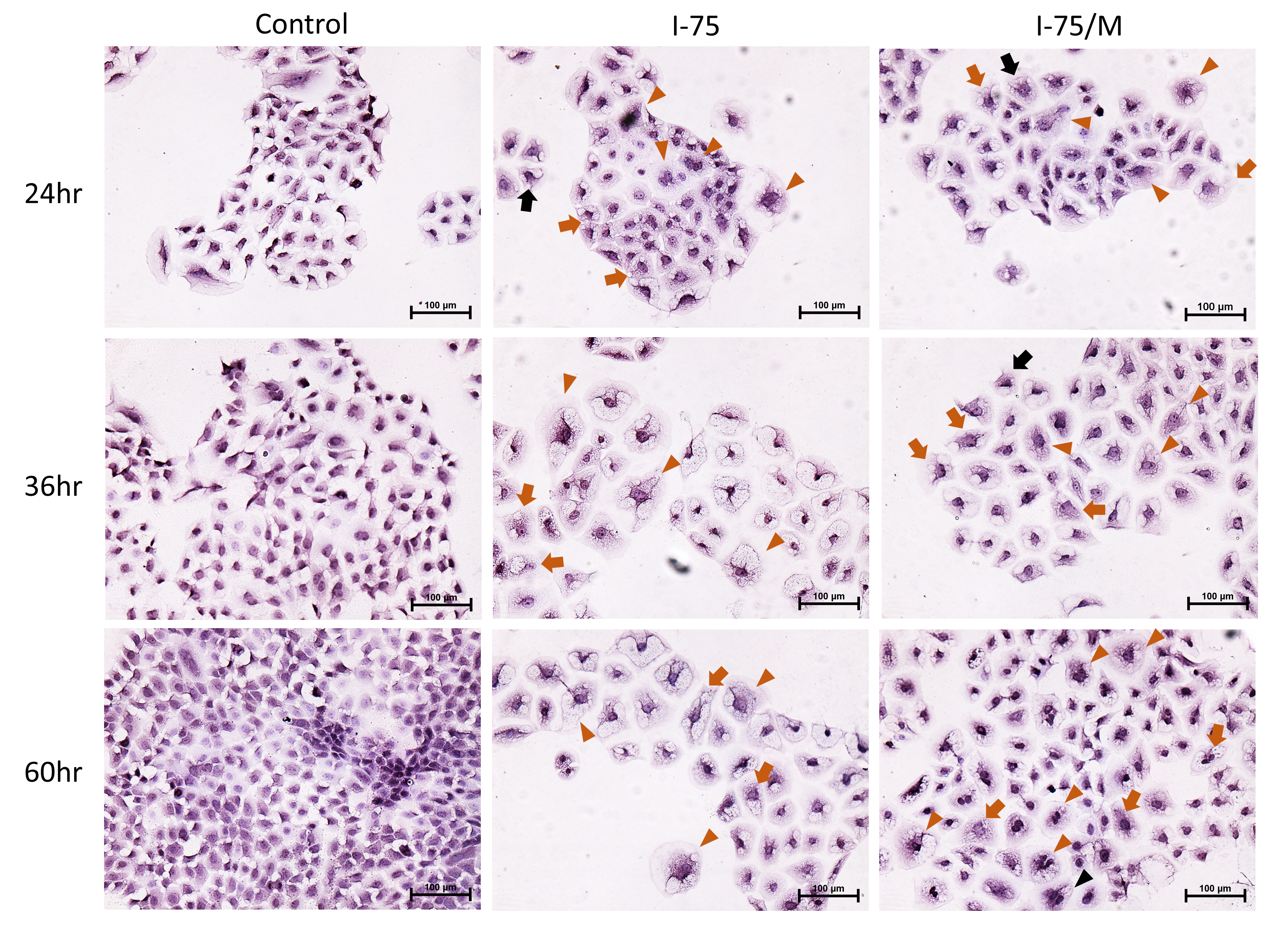

Supplement: Supplementary file 1 [file ijms-24-10945-s001.zip › SUPP.S1.CIN.NRK52E.tif]

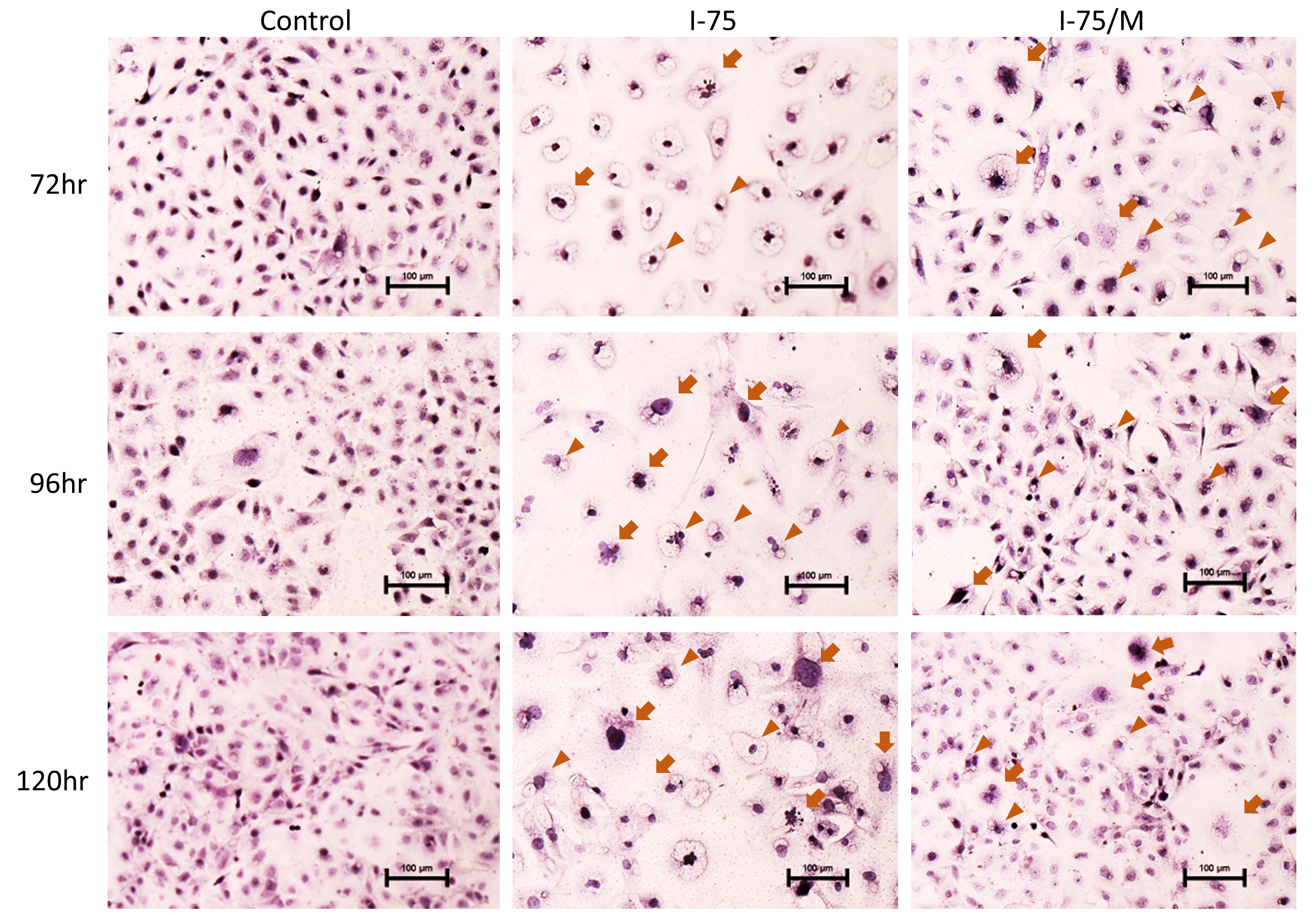

Supplement: Supplementary file 1 [file ijms-24-10945-s001.zip › SUPP.S2.CIN.HK2.72-120hr.tif]
